# Decision-making from temporally accumulated conflicting evidence: The more the merrier

**DOI:** 10.1167/jov.23.1.3

**Published:** 2023-01-04

**Authors:** Viola Mocz, Yaoda Xu

**Affiliations:** 1 Visual Cognitive Neuroscience Lab, Department of Psychology, Yale University, New Haven, CT, USA

**Keywords:** perceptual decision-making, numerosity judgment, ratio, discrete evidence

## Abstract

How do humans evaluate temporally accumulated discrete pieces of evidence and arrive at a decision despite the presence of conflicting evidence? In the present study, we showed human participants a sequential presentation of objects drawn from two novel object categories and asked them to decide whether a given presentation contained more objects from one or the other category. We found that both a more disparate ratio and greater numerosity of objects improved both reaction time (RT) and accuracy. The effect of numerosity was separate from ratio, where with a fixed object ratio, sequences with more total objects had lower RT and lower error rates than those with fewer total objects. We replicated these results across three experiments. Additionally, even with the total presentation duration equated and with the motor response assignment varied from trial to trial, an effect of numerosity was still found in RT. The same RT benefit was also present when objects were shown simultaneously, rather than sequentially. Together, these results showed that, for comparative numerosity judgment involving sequential displays, there was a benefit of numerosity, such that showing more objects independent of the object ratio and the total presentation time led to faster decision performance.

## Introduction

In everyday environments, we often face conflicting sensory input and need to retain sufficient sensory information before we can make a judgment. For example, in deciding the optimal lane to drive in during heavy traffic, we need to collect and retain sufficient sensory input from the lanes to our left and right before deciding which lane has faster moving traffic. How does the ratio of the two types of conflicting sensory information and the total amount of sensory information affect our performance? When the ratio is fixed, do we make better or worse decisions if we receive more sensory information? To address these questions, in this study, we asked human participants to perform a comparative numerosity judgment task by showing them a sequential presentation of objects from two distinctive object categories and then asking them to judge which category contained more objects in the sequence. We varied both the relative number (i.e., ratio) and the absolute number of the two types of objects shown in the sequence.

Tasks requiring participants to make a decision in the face of conflicting sensory input have been studied heavily in the context of decision making (Hanks & Summerfield, 2017). For example, in monkeys performing a decision making task determining whether an array of dots are primarily moving to the right or left, lateral intraparietal neurons have been implicated in calculating the decision, with neuronal activities ramping up during the delay period corresponding with sensory evidence accumulation (Shadlen & Newsome, 2001). The behavioral process of this form of decision-making has been modeled most often using drift diffusion, where evidence is continuously accumulated in a stochastic manner to reach a decision threshold (Forstmann, Ratcliff, & Wagenmakers, 2016; Gold & Shadlen, 2007; Ratcliff, Smith, Brown, & McKoon, 2016; O'Connell, Shadlen, Wong-Lin, & Kelly, 2018). In addition to using a continuously moving dot field in which a decision may be made at any time when enough evidence is accumulated, sequentially presented discrete events, similar to those used in our task, have also been used in perceptual decision-making tasks (e.g., De Lange, Jensen, & Dehaene, 2010; Brunton, Botvinick, & Brody, 2013; Morcos & Harvey, 2016). In such a paradigm, discrete chunks of sensory evidence are presented sequentially at a fixed rate, but the total amount of evidence shown could vary. Once all the evidence is presented, observers are asked to make a decision. Such a decision-making paradigm is related to, but somewhat different from, the traditional continuous decision-making paradigm. To better explain the complexity and detailed responses observed in various perceptual decision making tasks, recent studies have argued for more complex forms of the drift-diffusion model as well as entirely different kind of models that are non stochastic and are history dependent (e.g., Brunton et al., 2013; Morcos & Harvey, 2016; Pardo-Vazquez et al., 2019; Pirrone, Reina, & Gobet, 2021).

Although these studies have greatly enriched our understanding of various aspects of perceptual decision-making, our understanding remains incomplete regarding how the ratio of the two types of conflicting sensory information and the total amount of sensory information received would impact performance. De Lange et al. (2010) presented human participants a stream of one to five arrows that pointed either left or right, and asked them to decide whether there were more left or right arrows in the stream. Performance was faster on trials with more arrows and on trials in which more evidence favored one direction over the other. However, because the ratio and the number of arrows shown were not manipulated separately, how these two factors may contribute to performance remains unknown. Brunton et al. (2013) found that, given the same ratio and the same total number of audio clicks from two sound locations, longer presentation duration of each click resulted in rats more accurately localizing the location that emitted more clicks. This finding suggests that, with a fixed ratio, receiving more sensory information could lead to greater performance accuracy. Receiving a greater amount of sensory information can come from extending the presentation duration of each discrete event (e.g., longer clicks), or having more discrete events carrying the same information (e.g., more shorter clicks). It is presently unknown whether or not both types of manipulations would have the same impact on performance.

In the number literature, when two sets of objects are presented side by side and people need to decide which set has more objects, numerosity judgment follows Weber's law, such that the perceived difference between the two sets is proportional to the number of objects contained in a set, with larger sets requiring a greater difference between the sets to produce the same numerosity judgment accuracy (Weber, 1834; Weber, 1978; Jevons, 1871; Ross, 2003). In other words, as long as the object ratio for the two sets is kept constant, similar precision in judgment can be obtained. Weber's law in enumeration can also been seen when two to three sets of objects are intermixed and presented simultaneously and observers have to enumerate the sets in parallel (Halberda, Sires, & Feigenson, 2006; Poltoratski & Xu, 2013; Liu, Zheng, Huang, & Cicchini, 2020). Weber's law applies beyond numerosity judgments and captures a more general principle between the perceived difference and the actual physical difference of the input stimulus in comparative judgment tasks across different input modalities.

Because our task involves comparing the numerosity of objects from two categories, according to Weber's law, performance may only be affected by the ratio but not by the total number of objects shown. That is, as long as the object ratio is kept constant, performance may not be affected by the number of objects shown.

The computational mechanisms behind Weber's law have long puzzled researchers. It is noted that, although Weber's law addresses performance accuracy, it does not explicitly address reaction time (RT). In a recent study (Pardo-Vazquez, et al., 2019), rats performed a sound intensity discrimination task that varied both the ratios and average magnitudes of the two sounds to be discriminated against. It was found that, although accuracy did not change for the same intensity ratio, the RT was faster when the intensity was higher. This finding would predict that, in our task, with the same object ratio but varying number of objects, accuracy may not vary, but the RT would be faster when more objects are shown in a sequence.

It is also possible that people may not track the ratio between the two object sets, but rather just the difference in their numerosity. Consequently, performance may not be determined by ratio, but only by the absolute difference between the two sets in the sequence. This finding is more aligned with the prediction of the drift-diffusion model mentioned earlier in this article, whereby the decision response is largely determined by the amount of evidence accumulated in favor of one or the other choice options. Although this does not apply to simultaneously presented visual arrays as shown by Weber's law (whereby a greater absolute difference is needed between two sets to achieve the same accuracy when the numerosity is large), in a temporal comparative numerosity judgment task, however, this remains a possibility.

In our task, because sensory information needs to be retained for a prolonged period of time before a decision can be made, limitations in attention and, particularly, visual working memory (VWM), may impact performance. Ample behavioral studies from the past few decades have shown that VWM has a limited capacity (e.g., Philips, 1974; Pashler, 1988; Luck & Vogel, 1997). Such a capacity limitation is not only present in simultaneously presented visual arrays, but is also observed when items are shown sequentially one at a time (e.g., Xu & Chun 2006). Limited attentional resources and VWM capacity would predict that, when more objects are shown in a sequence in our task, attention and VWM may be more heavily taxed, leading to a decreased performance.

The current literature thus does not provide a clear prediction regarding how object ratio and the total amount of sensory information exposure would impact comparative numerosity judgment. Additionally, previous studies have largely involved sensory stimuli, such as dot motion and sound intensity, leaving it unknown whether information accumulation and comparison involving more abstract visual information, such as object categories, may follow the same response patterns. To address these questions, in this study, adapting the paradigms from Brunton et al. (2013) and De Lange et al. (2010) (see also Morcos & Harvey, 2016), we presented human participants different exemplars from two novel object categories from Op de Beeck, Baker, DiCarlo, and Kanwisher (2006) at fixation and asked them to decide which of the two types of objects were more numerous in a trial. We manipulated the ratio between the two object categories and the total number of objects in a sequence. Because the total amount of time in a sequence can also be a confounding variable in decision-making, we further manipulated the time each object was shown to compare the performance on trials with the same total presentation duration and same object ratio, but a different total number of objects. To understand whether the effects we obtained were specific to sequentially presented objects, we also examined performance when all objects were shown simultaneously in a spatial array.

## Experiment 1: Temporal sequences with varying ratio and numerosity

In this experiment, we wanted to examine how the ratio and numerosity of two object categories presented in a temporal sequence would affect people's RT and accuracy when making a comparative numerosity judgment (i.e., deciding which of the two objects is more numerous in a sequence).

### Design and procedures

#### Participants

A total of 12 participants took part in this experiment (mean age, 24.83 ± 2.36 years; eight females; nine right handed). The number of participants included in this and all subsequent experiments was determined by the number of subjects included in other similar published studies in decision-making and numerosity judgment (e.g., *N* = 5 in Ross [2003], *N* = 14 in De Lange et al. [2010], *N* = 7 in Cicchini et al. [2016], *N* = 16 per experiment in Ratcliff et al. [2018], and *N* = 7 in Pomè et al. [2019]). All had normal or corrected-to-normal vision and were paid for their participation. An additional two participants also took part in the experiment, but were excluded for not completing the entire task and for a chance level performance accuracy, respectively. The experiment was approved by the Yale University Institutional Review Board for human studies. All participants provided informed written consent prior to the start of the experiment.

#### Stimuli and design

**Figure 1.**
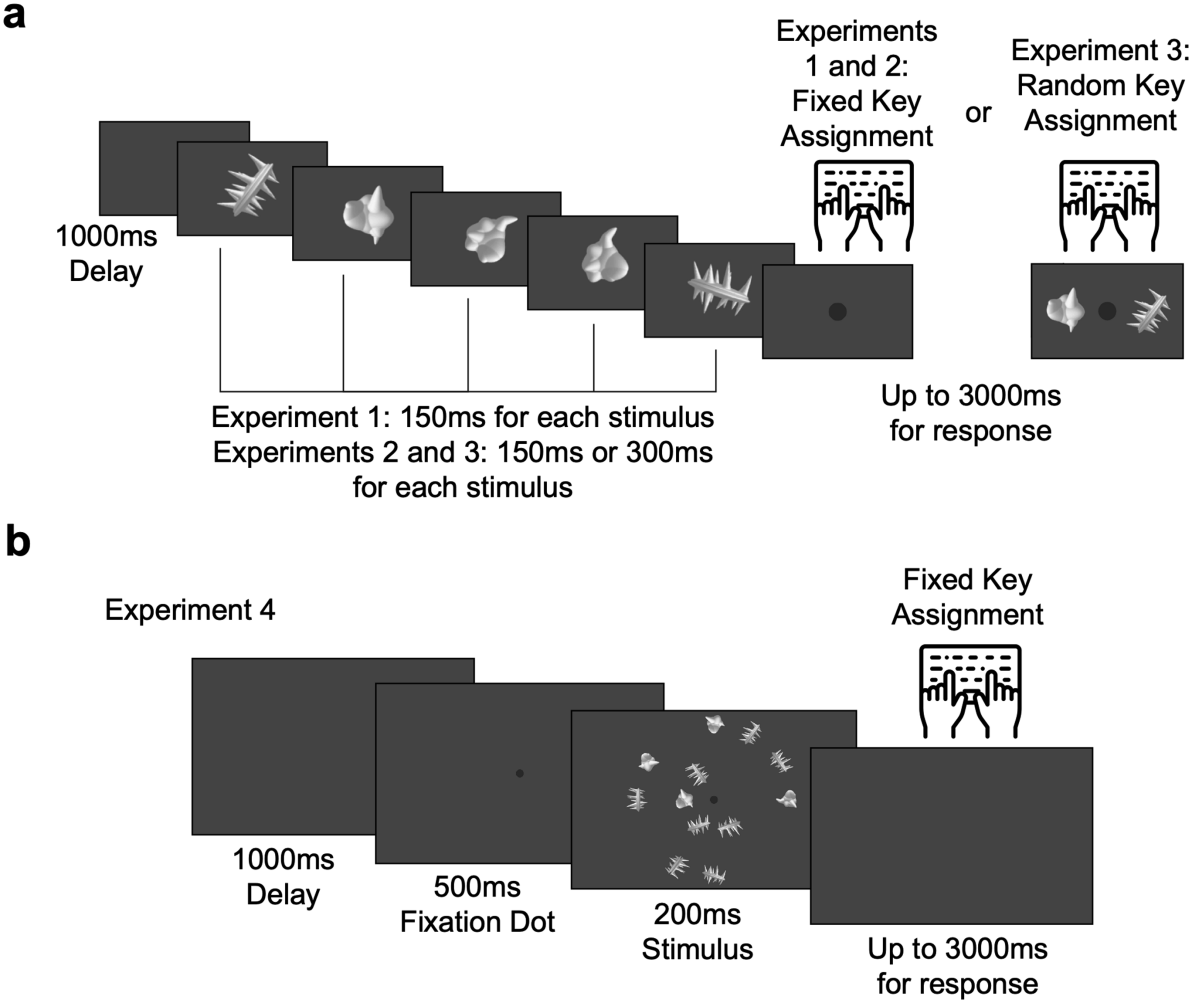
Example trial sequence and the factors manipulated in each experiment. (a) In Experiments 1 through 3, objects from two novel object categories, the smoothies and the spikies, were presented at fixation in a randomly intermixed order. Participants had to decide whether a given sequence contained more spikies or smoothies. Across the experiments, the ratio between the two object sets (1:2 and 2:3), the size of the smaller of the two object sets (2, 4, and 6), and the presentation duration (150 ms or 300 ms) were manipulated. Although the response key assignment was fixed for each participant in Experiments 1 and 2, it varied from trial to trial in Experiment 3, with the key corresponding to each object category shown at the end of a trial. (b) In Experiment 4, objects from the same two object categories were presented all at once along the perimeter of two invisible concentric circles. Other aspects of this experiment were identical to those of Experiment 1.

In this experiment, participants viewed in each trial a sequential presentation of exemplar objects from two novel object categories. They then judged which one of the two categories had more objects shown (Figure 1). We used the smoothy and spiky objects from Op de Beeck, et al. (2006) and chose 10 different exemplar objects from each of these two object categories with varying identities and orientations as in a prior study (Vaziri-Pashkam & Xu, 2019). Each object was shown at fixation for 150 ms on a grey background at a size of about 10° × 10° of visual angle. Participants pressed one of the two response keys (F or J) to indicate their responses, with the mapping between the response keys and the object categories fixed for each participant and counterbalanced across participants. The participants had 3 seconds to respond. After a 1-second intertrial interval, the next trial would start.

To test how the relative (i.e., ratio) and the absolute number of objects shown from the two object categories would impact performance, the ratio between the two objects was set to be either 1:2 or 2:3, with the size of the smaller object set to be either 2, 4, or 6. This resulted in a total of six possible trial conditions (2 ratios x 3 sizes for the smaller object set). For the 1:2 ratio, the conditions were 2 versus 4, 4 versus 8, and 6 versus 12. For the 2:3 ratio, the conditions were 2 versus 3, 4 versus 6, and 6 versus 9. Within a trial, the presentation order for the two types of objects was completely random. We also made sure that a given object exemplar did not immediately repeat when 12 objects were shown in trials from the 6 versus 12 condition because there were only 10 unique exemplars from each object category. The presentation order for the different trial conditions were randomly intermixed, with the chance of objects from either category appearing more numerous in a trial being 50%. Results were averaged between the responses from the two object categories.

Before the start of the experiment, the participants completed a practice session with 12 trials. They could move on to the main experiment if they reached at least 75% accuracy. If not, they would go through another practice session with 12 trials. All the participants were able to achieve at least 75% accuracy by the end of the second practice session. The main experiment consisted of 480 trials, with 80 trials for each condition, and these trials were distributed evenly across 10 trial blocks. Both RT and accuracy for each trial were recorded. The whole experiment lasted approximately 50 minutes.

#### Analysis

For each participant, trials whose RTs were 3 standard deviations above the mean of that participant were considered as outliers and were removed from further analyses. This resulted in the removal of 1.45% of the total data from the experiment. The means of the RT and error rate for each trial condition were then calculated for each participant and the results were analyzed at the group level. The analysis for RT only included correctly responded trials. A repeated measures analysis of variance (ANOVA) including ratio and numerosity as factors was conducted to test the impact of these two factors and their interaction on performance. Post hoc pairwise *t*-tests were also conducted to further understand the effect of numerosity, with all *p* values corrected for multiple comparisons using the Benjamini–Hochberg method (Benjamini & Hochberg, 1995). For the ANOVA results, we calculated effect size using a partial eta-squared measure (Cohen, 1973), since it is a less biased measure of effect size for ANOVAs that is comparable across study designs (Richardson, 2011). It is defined as:

$$\begin{array}{c} {\eta^{2}}_{P}=\frac{SS_{effect}}{SS_{effect}+SS_{s/cells}} \end{array}$$

where *SS*_effect_ is the sum of squares of the effect of interest and *SS*_s/cells_ is the sum of squares for within-subject error. For the pairwise *t*-tests, we calculated effect size using Cohen's D (Cohen, 1969; Cohen, 1988).

### Transparency and openness

For this experiment and all other experiments hereafter, we report how we determined any data exclusions, all manipulations, and all measures in the study. Data and analysis code are available on OSF at this link: https://osf.io/4rgyv/. All statistical tests were conducted using R (R Core Team, 2018). This study's design and its analysis were not preregistered.

### Results and discussion

**Figure 2.**
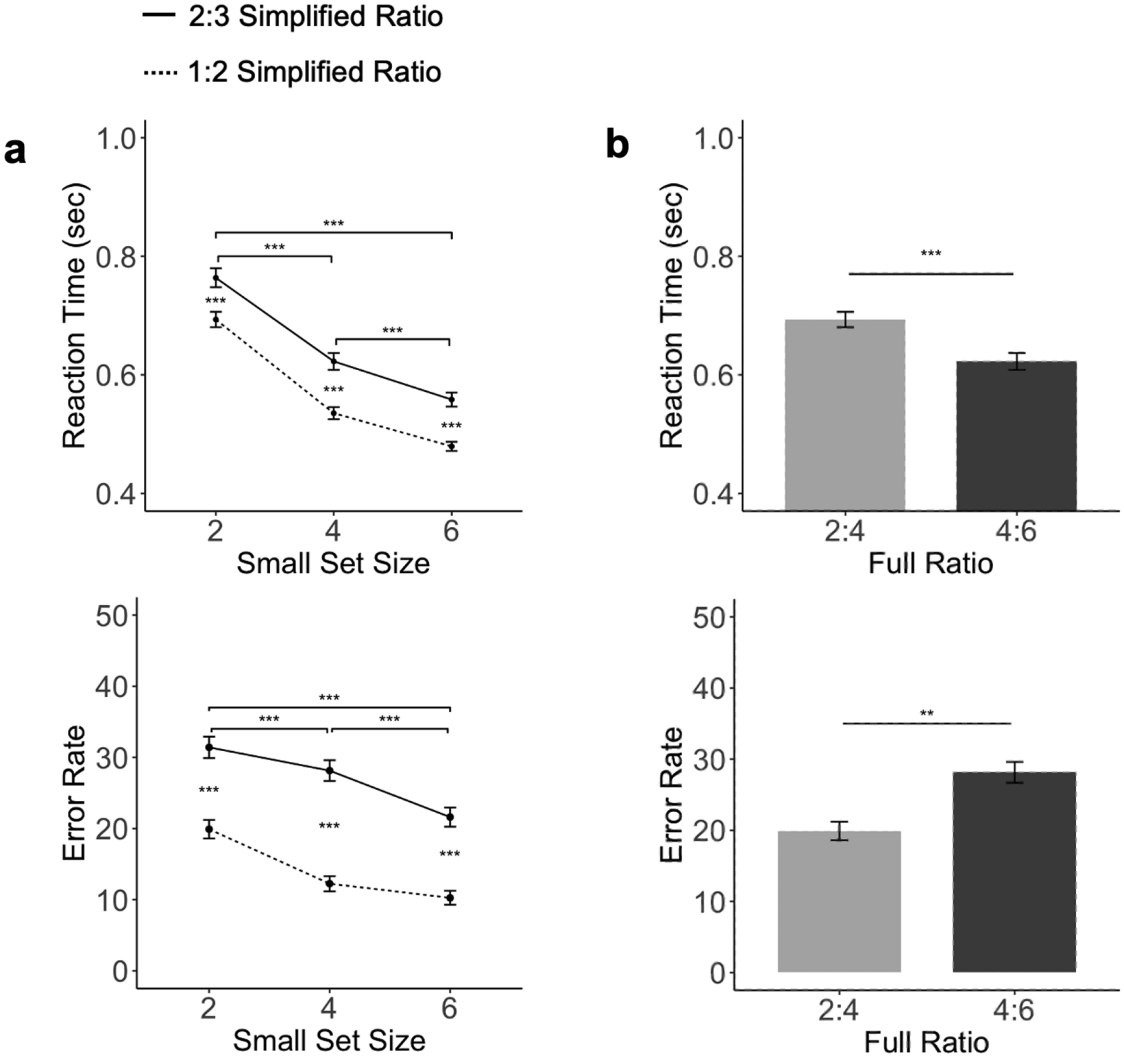
Results of Experiment 1. (a) RTs and error rates as a factor of ratio and the size of the smaller object set. (b) RTs and error rates when the absolute difference between the two object sets was fixed, comparing 2 versus 4 (at a ratio of 1:2) and 4 versus 6 (at a ratio of 2:3). The error bar represents ±1 standard error. **p < 0.01, ***p < 0.001, all corrected for multiple comparisons using the Benjamini–Hochberg method.

We measured both the RT and error rates and the results are plotted by numerosity (i.e., the number of objects shown in the small set) (Figure 2a) and by the object ratio (Figure 2b). Also, see the mean and standard deviation for both RT and error rates for each full object ratio in Table 1.

**Table 1.** Results of Experiment 1. The mean (*M*) and standard deviations (SD) of RTs and error rates for each ratio (*N* = 12).

Full ratio	RT (*M*, s)	RT (SD, s)	Error rate (*M*, percent)	Error rate (SD, percent)
2:3	0.764	0.402	31.404	46.438
2:4	0.693	0.355	19.893	39.941
4:6	0.623	0.369	28.132	44.988
4:8	0.535	0.293	12.238	32.790
6:9	0.558	0.325	21.602	41.174
6:12	0.479	0.224	10.251	30.347

For the RT measures, a repeated measure ANOVA showed a main effect of ratio, with RTs being faster for a more disparate ratio, that is, faster responses for the 1:2 than the 2:3 ratio, *F*(1,11) = 12.31, *p* < 0.01, η^2^_*P*_ = 0.53. Interestingly, there was also a main effect of numerosity independent of ratio, with RTs being faster when more objects were shown in a trial, *F*(2,22) = 33.77, *p* < 0.001, η^2^_*P*_ = .75 (Figure 2a). Post hoc paired *t*-tests further confirmed this finding, with differences between trial pairs with the same ratio but different numerosity (e.g., between 2 vs. 3 and 4 vs. 6) all reaching significance, *t*s > 5.33, *p*s < 0.001, *d*s > 0.77. There was no interaction between ratio and numerosity, F(2,22) = 0.60, *p* = 0.55, η^2^_*P*_ = 0.05.

To understand if a difference in ratio still played a role when the absolute difference between the two sets was fixed, we also compared RT differences between 2 versus 4 (at a ratio of 1:2) and 4 versus 6 (at a ratio of 2:3). We found that RT was significantly faster in the 4 versus 6 condition than in the 2 versus 4 condition, *t*(11) = 4.55, *p* < 0.001, *d* = 1.31 (Figure 2b).

Similar to the RT results, in error rate measure, a repeated measure ANOVA also showed a main effect of ratio, with error rates being lower for a more disparate ratio, *F*(1,11) = 114.76, *p* < 0.001, η^2^_*P*_ = 0.91, and, once again, a main effect of numerosity, with error rates being lower when more objects were included in a trial, *F*(2,22) = 18.24, *p* < 0.001, η^2^_*P*_ = 0.62 (Figure 2a). Post hoc paired *t*-tests further confirmed this finding, *t*s > 3.85, *p*s < 0.001, *d*s > 0.55 (corrected), with error rate always being lower when more objects were shown for trials with the same object ratio. There was an interaction between ratio and numerosity, *F*(2,22) = 4.06, *p* = 0.031, η^2^_*P* _ = 0.27, with the effect of ratio being greater when four, *t*(23) = 8.14, *p* < 0.001, *d* = 1.66, rather than two, *t*(23) = 7.71, *p* < 0.001, *d* = 1.57, or six, *t*(23) = 7.41, *p* < 0.001, *d* = 1.51, objects were shown in the small object set.

As in the RT analysis, we also assessed the effect of ratio when the absolute difference between the two sets was fixed to 2. Here we found that 2 versus 4 had a lower error rate than 4 versus 6, *t*(11) = 3.42, *p* < 0.01, *d* = 0.98. This was the opposite as the effect found in RTs, likely indicating a speed–accuracy tradeoff.

Overall, both the relative number (ratio) and the absolute number (numerosity) of objects included in a temporal sequence affected participants’ speed and accuracy in comparative number decision. Such an effect of numerosity was not expected from Weber's law. Additionally, when the absolute difference between two sets was fixed to 2, a more disparate ratio resulted in a slower RT but higher accuracy. Note that here we have the effects of ratio and numerosity pitted against each other: the high ratio set had fewer total number of objects than the low ratio set. The existence of a speed–accuracy tradeoff, rather than an overwhelming effect of either on both RT and accuracy, suggests that both ratio and numerosity play similar roles in determining performance here.

It could be argued that trials with more objects took longer time to present and process. Perhaps a prolonged exposure time to the same amount of information could lead to faster and more accurate performance. Experiment 2 was conducted to test this possibility.

## Experiment 2: Temporal sequences with fixed presentation time

To understand how the total amount of stimulus presentation time may affect performance, in this experiment, while keeping both the total stimulus presentation duration and object ratio constant, we investigated whether or not numerosity would still affect comparative numerosity judgment in a temporal sequence.

### Design and procedures

#### Participants

A total of 12 new participants took part in Experiment 2 (mean age, 22.5 ± 3.39 years; 10 females; 10 right-handed).

#### Stimuli and design

The design of this experiment was similar to that of Experiment 1 in that the ratio between the two objects was still either 1:2 or 2:3. However, it differed from Experiment 1 in two aspects: first, the size of the smaller set could only be either 2 or 4; and second, the presentation duration for each object in a trial sequence could be either 150 ms or 300 ms (Figure 1a). This led to a total of 8 different trial conditions (2 ratios × 2 durations × 2 sizes for the smaller object set) and they were: for the 1:2 ratio, 2 versus 4 at 150 ms, 2 versus 4 at 300 ms, 4 versus 8 at 150 ms, and 4 versus 8 at 300 ms; and for the 2:3 ratio, 2 versus 3 at 150 ms, 2 versus 3 at 300 ms, 4 versus 6 at 150 ms, and 4 versus 6 at 300 ms. This enabled us to not only replicate the results from Experiment 1, but also more critically compare two pairs of trials with the same total presentation duration and ratio but different numerosity. The first pair of such trials were the 2 versus 4 at 300 ms and the 4 versus 8 at 150 ms trials, and the second pair of such trials were the 2 versus 3 at 300 ms and the 4 versus 6 at 150 ms trials. The main experiment consisted of 400 trials, with 50 trials for each condition. All trials with the same presentation duration were grouped together. Within each presentation duration, trials from the different conditions were randomly intermixed and evenly distributed into five trial blocks. Trial blocks with the two presentation durations were shown in an alternate fashion, with the presentation duration of the first block further balanced across participants.

#### Analysis

Following the same procedure as described in Experiment 1, a total of 1.63% of the data were removed as outliers from further analysis. Repeated measures ANOVAs including ratio, the size of the smaller object set, and presentation duration as factors were conducted to analyze the RTs of the correctly responded trials and the error rates. Further pairwise *t*-tests were conducted on pairs of trials with the same ratio and presentation duration but different numerosity. All other details of the experiment were identical to those of Experiment 1.

### Results and discussion

**Figure 3.**
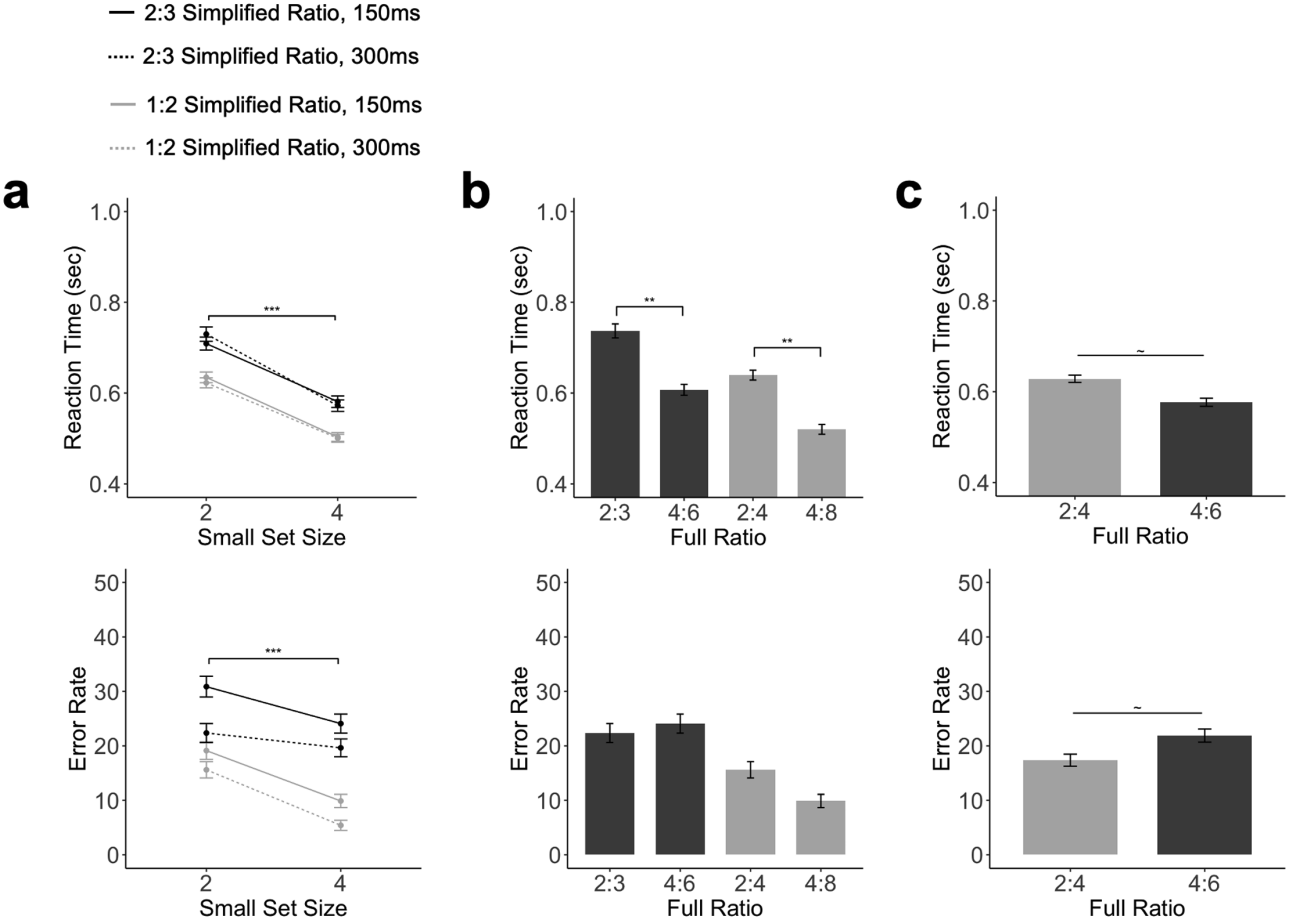
Results of Experiment 2. (a) RTs and error rates as a factor of ratio, the size of the smaller object set, and stimulus presentation duration. (b) RT and error rate for trial pairs with the same ratio and total presentation duration, comparing the pair 2:4 at 300 ms and 4:8 at 150 ms, and the pair 2:3 at 300 ms, and 4:6 at 150 ms (c) RTs and error rates when the absolute difference between the two object sets was fixed, comparing 2 versus 4 (at a ratio of 1:2) and 4 versus 6 (at a ratio of 2:3). The error bar represents ±1 standard error. **p < 0.01, ***p < 0.001, all corrected for multiple comparisons using the Benjamini–Hochberg method.

As in Experiment 1, we measured both RT and error rates and the results are plotted by the number of objects shown in the small set (Figure 3a) and by the object ratio (Figures 3b and 3c). Also, see the mean and standard deviation for both RT and error rates for each full object ratio and stimulus duration in Table 2.

**Table 2.** Results of Experiment 2. The mean (*M*) and standard deviations (SD) of RTs and error rates for each ratio and stimulus duration (*N* = 12).

Full ratio	Stimulus duration (ms)	RT (*M*, s)	RT (SD, s)	Error rate (*M*, percent)	Error rate (SD, percent)
2:3	150	0.709	0.291	30.860	46.230
2:3	300	0.729	0.332	22.359	41.702
2:4	150	0.634	0.257	19.120	39.358
2:4	300	0.622	0.244	15.593	36.309
4:6	150	0.581	0.271	24.080	42.792
4:6	300	0.572	0.275	19.627	39.751
4:8	150	0.503	0.225	9.866	29.845
4:8	300	0.500	0.207	5.396	22.613

Replicating the results of Experiment 1, a repeated measures ANOVA showed that RT was faster both for a more disparate ratio, *F*(1,11) = 16.25, *p* < 0.01, η^2^_*P*_ = 0.59, and when more objects were shown in a trial, *F*(1,11) = 57.42, *p* < 0.001, η^2^_*P*_  = 0.84 (Figure 3a). There was no main effect of presentation duration, *F*(1,11) = 0.018, *p* = 0.89, η^2^_*P*_ = 0.001. There was no two-way interactions between numerosity and ratio, *F*(1,11) = 0.64, *p* = 0.44, η^2^_*P*_ = 0.05, between numerosity and presentation duration, *F*(1,11) = 1.19, *p* = 0.29, η^2^_*P*_ = 0.09, or between ratio and presentation duration, *F*(1,11) = 1.87, *p* = 0.19, η^2^_*P*_ = 0.14. There was a three-way interaction among all three factors, *F*(1,11) = 6.39, *p* < 0.05, η^2^_*P*_ = 0.37.

When comparing the RTs of the two pairs of trials with the same object ratio and total presentation duration but different numerosities, we found that when combining both cases, trials with more objects had a significantly faster RT, *t*(11) = 4.75, *p* < 0.001, *d* = 1.37 (Figure 3b). These results were further confirmed when each pair was analyzed separately, *t*s > 4.19, *p*s < 0.01, *d*s > 1.20 (corrected). Thus, even when total trial duration was equated, object numerosity still impacted people's decision-making speed.

After Experiment 1, to understand if a difference in ratio still played a role when the absolute difference between the two sets was fixed, we also compared RT differences between 2 versus 4 (at a ratio of 1:2) and 4 versus 6 (at a ratio of 2:3). We found that there was no significant difference in RT between the 2 versus 4 and 4 versus 6 conditions, *t*(11) = 1.99, *p* = 0.071, *d* = 0.57 (Figure 3c).

As in Experiment 1, in error rate measures, we similarly observed an effect of ratio, *F*(1,11) = 82.96, *p* < 0.001, η^2^_*P*_ = 0.88, and numerosity, *F*(1,11) = 32.34, *p* < 0.001, η^2^_*P*_ = 0.74 (Figure 3a), with error rates being lower for trials with a more disparate object ratio and when more objects were shown. Unlike the RT measure, there was a main effect of presentation duration, where error rate was lower under a longer presentation duration, *F*(1,11) = 8.89, *p* < 0.05, η^2^_*P*_ = 0.44. There was no two-way interaction between numerosity and ratio, *F*(1,11) = 3.60, *p* = 0.08, η^2^_*P*_ = 0.24, between numerosity and presentation duration, *F*(1,11) = 0.43, *p* = 0.52, η^2^_*P*_ = 0.03, or between the ratio and presentation duration, *F*(1,11) = 1.14, *p* = 0.31, η^2^_*P*_ = 0.09, and no three-way interaction among all three factors, *F*(1,11) = 1.15, *p* = 0.30, η^2^_*P*_ = 0.09.

When comparing the error rate of trials with the same total presentation duration and ratio of objects but different numerosities, we found no effect of numerosity on the error rate, *t*(11) = 0.73, *p* = 0.48, *d* = 0.21, and this was confirmed for each pair separately, *t*s < 1.98, *p*s > 0.14, *d*s < 0.57 (corrected) (Figure 3b).

As in the RT analysis, we also examined the effect of ratio when the absolute difference between the two sets was fixed. We found that when the absolute difference between the two sets was fixed to 2, there was no significant difference in error rate between the 2 versus 4 and 4 versus 6 conditions, *t*(11) = 2.03, *p* = 0.066, *d* = 0.58 (Figure 3c).

Overall, these results replicated those from Experiment 1 and showed that both the relative number (ratio) and the absolute number (numerosity) of objects included in a temporal sequence affected participants’ speed and accuracy in comparative number judgment. Moreover, when object ratio and the total presentation duration were equated, we still found a strong effect of object numerosity in RT. The same effect in error rate was much attenuated. When the absolute difference between the two sets was fixed to 2, there was no effect of ratio on RT nor accuracy. This again suggests similar effects of ratio and numerosity when the two effects were pitted against each other.

## Experiment 3: A replication with randomized key mappings

One could argue that in Experiments 1 and 2, because the response key assignments were fixed for the entire experiment, participants could potentially prepare their motor responses early in some of the trials when more objects were shown, leading to faster RTs for those trials. To examine this possibility, in this experiment we replicated Experiment 2, but with the key assignment changed randomly from trial to trial and only given after the presentation of all the objects in a trial.

### Design and procedures

#### Participants

A total of 12 participants took part in this experiment (mean age, 23.58 ± 3.42 years; 8 females; all right handed). An additional participant also took part in the experiment, but was excluded for not completing the entire task. Of the 12 participants, 3 also participated in Experiment 1 and another 3 participated in Experiment 2.

#### Stimuli and design

The design of this experiment was almost identical to that of Experiment 2. The only difference was that, after all the objects were shown for a given trial, two objects, one from each category, appeared on the two sides of the screen. Participants were asked to press the F key if more objects in the trial came from the left object category and to press the J key if the reverse was true. The sides on which the two objects appeared changed randomly from trial to trial and were counterbalanced within a block of trials.

#### Analysis

Following the same procedure as described in Experiment 1, a total of 1.38% of the data were removed as outliers from further analysis.

All other details of the experiment were identical to those of Experiment 2.

### Results and discussion

**Figure 4.**
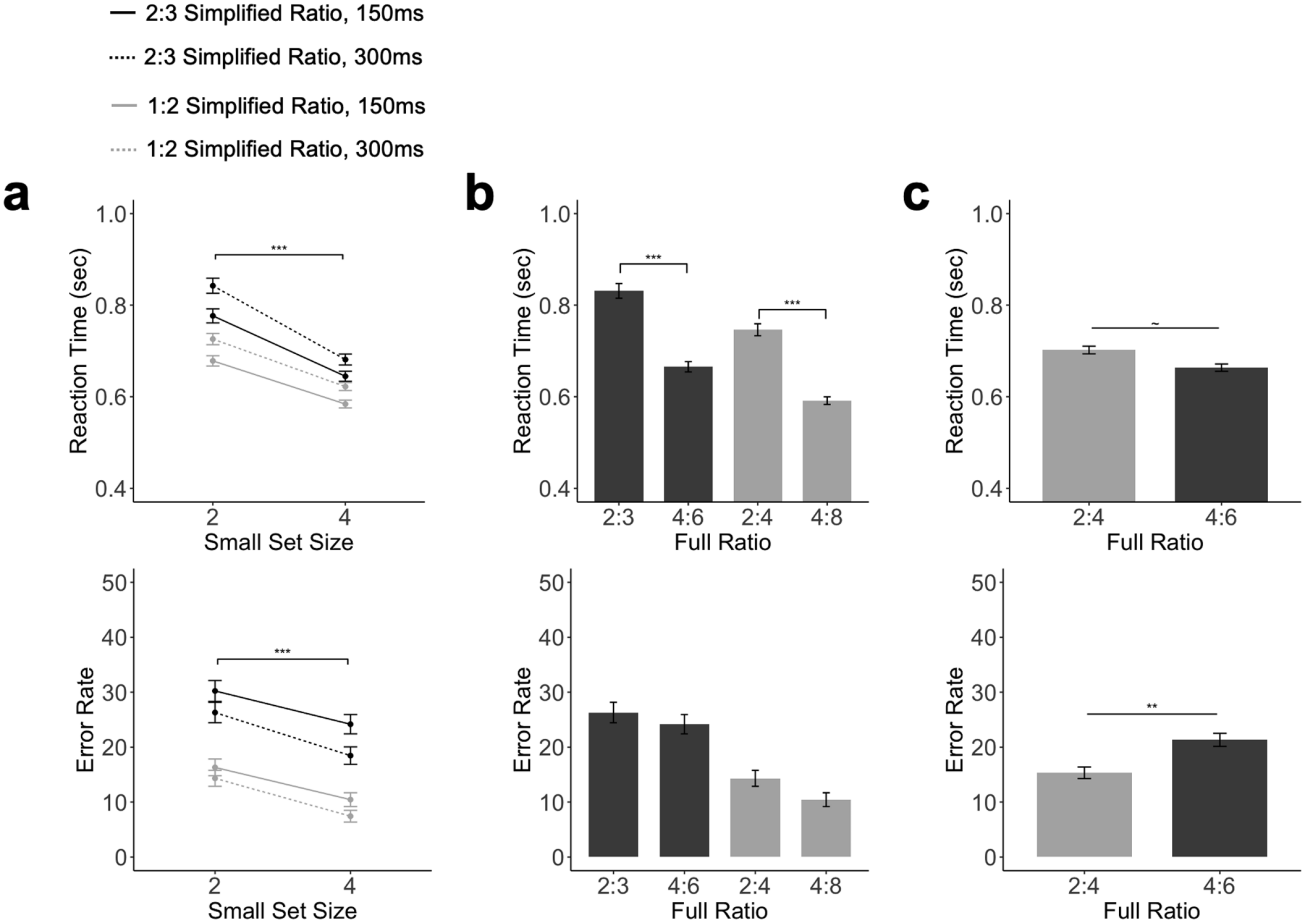
Results of Experiment 3. **(a)** RTs and error rates as a factor of ratio, the size of the smaller object set, and stimulus presentation duration. (b) RTs and error rates for trial pairs with the same total presentation duration, comparing the pair 2:4 at 300 ms and 4:8 at 150 ms, and the pair 2:3 at 300 ms, and 4:6 at 150 ms. (c) RTs and error rates when the absolute difference between the two object sets was fixed, comparing 2 versus 4 (at a ratio of 1:2) and 4 versus 6 (at a ratio of 2:3). The error bar represents ±1 standard error. **p < 0.01, ***p < 0.001, all corrected for multiple comparisons using the Benjamini–Hochberg method.

As before, we measured both the RT and error rates, and the results are plotted by the number of objects shown in the small set (Figure 4a) and by the object ratio (Figures 4b and 4c). Also, see the mean and standard deviation for both RT and error rates for each full object ratio and stimulus duration in Table 3.

**Table 3.** Results of Experiment 3. The mean (*M*) and standard deviations (SD) of RTs and error rates for each ratio and stimulus duration (*N* = 12).

Full ratio	Stimulus duration (ms)	RT (*M*, s)	RT (SD, s)	Error rate (*M*, percent)	Error rate (SD, percent)
2:3	150	0.776	0.313	30.220	45.960
2:3	300	0.842	0.339	26.287	44.058
2:4	150	0.678	0.252	16.302	36.969
2:4	300	0.725	0.273	14.310	35.048
4:6	150	0.644	0.229	24.161	42.841
4:6	300	0.681	0.264	18.443	38.816
4:8	150	0.583	0.195	10.437	30.600
4:8	300	0.622	0.203	7.419	26.231

Replicating the results of Experiments 1 and 2, we again found that RT was significantly faster both for a more disparate ratio, *F*(1,11) = 32.82, *p* < 0.001, η^2^_*P*_ = 0.74, and when more objects were included in a trial, *F*(1,11) = 28.63, *p* < 0.001, η^2^_*P*_ = 0.72 (Figure 4a). Unlike in Experiment 2, where we did not see a main effect of presentation duration in RT, here the longer presentation duration led to a slower RT, *F*(1,11) = 13.44, *p* < 0.01, η^2^_*P*_ = 0.55. There was also an interaction between ratio and object numerosity, *F*(1,11) = 10.05, *p* < 0.01, η^2^_*P*_ = 0.47. Pairwise *t*-tests revealed that, when ratio was fixed, the RT was faster when object numerosity was high, *t*s > 6.44, *p*s < 0.001, *d*s > 1.31 (corrected), but the effect seemed to be somewhat more pronounced for the 2:3 ratio (*d* = 1.33) than the 1:2 ratio (*d* = 1.31). There was no two-way interaction between numerosity and presentation duration, *F*(1,11) = 1.03, *p* = 0.33, η^2^_*P*_ = 0.08, or between ratio and presentation duration, *F*(1,11) = 0.96, *p* = 0.34, η^2^_*P*_ = 0.08, and no three-way interaction among all three factors, *F*(1,11) = 0.46, *p* = 0.51, η^2^_*P*_ = 0.04.

As in Experiment 2, when comparing the RTs of the two pairs of trials with the same object ratio and total presentation duration but different numerosities, we found that when combining both cases, trials with more objects had a significantly faster RT, *t*(11) = 5.10, *p* < 0.001, *d* = 1.47. These results were further confirmed when each pair was analyzed separately, *t*s > 4.50, *p*s < 0.001, *d*s > 1.30 (Figure 4b).

As before, to understand if a difference in ratio still played a role when the absolute difference between the two sets was fixed, we also compared RT differences between 2 versus 4 (at a ratio of 1:2) and 4 versus 6 (at a ratio of 2:3). We found that there was no significant difference in RT for the 2 versus 4 and 4 versus 6 conditions, *t*(11) = 2.02, *p* = 0.068, *d* = 0.58 (Figure 4c), although there was a trend of a more disparate ratio resulting in a slower RT, similar to the results from Experiments 1 and 2.

As in Experiment 2, in error rate measures, we observed both an effect of ratio, with a lower error rate for a more disparate ratio, *F*(1,11) = 299.29, *p* < 0.001, η^2^_*P*_ = 0.96, and an effect of numerosity, with lower error rate when more objects were shown in a trial, *F*(1,11) = 21.17, *p* < 0.001, η^2^_*P*_ = 0.65 (Figure 4a). There was no significant effect of stimulus duration on error rate, *F*(1,11) = 4.37, *p* = 0.06, η^2^_*P*_ = 0.28. There was no two-way interaction between numerosity and ratio, *F*(1,11) = 0.06, *p* = 0.81, η^2^_*P*_ = 0.005, between numerosity and presentation duration, *F*(1,11) = 0.16, *p* = 0.69, η^2^_*P*_ = 0.014, or between ratio and presentation duration, *F*(1,11) = 1.19, *p* = 0.29, η^2^_*P*_ = 0.09, and no three-way interaction among all three factors, *F*(1,11) = 0.006, *p* = 0.94, η^2^_*P*_ = 0.0005.

When comparing the error rate of trials with the same total presentation duration and ratio of objects but different numerosities, we found no effect on the number of objects on accuracy, *t*(11) = 1.45, *p* = 0.176, *d* = 0.42, and this was confirmed for each pair separately (*t*s < 1.72, *p*s > 0.22, *d*s < 0.49 (corrected) (Figure 4b).

As in the RT analysis, we also examined the effect of ratio when the absolute difference between the two sets was fixed. We found that when the absolute difference between the two sets was fixed to 2, 2 versus 4 had a lower error rate than 4 versus 6, *t*(11) = 3.92, *p* < 0.01, *d* = 1.13 (Figure 4c).

Overall, these results replicated those of Experiments 1 and 2 by showing that both the relative number (ratio) and the absolute number (numerosity) of objects included in a temporal sequence affected participants’ speed and accuracy in comparative number judgment. Moreover, these results showed that, even when total trial duration was equated and when participants could not prepare their motor responses earlier in the trials, we still found a strong effect of object numerosity in RT. The effect on the error rate was much weaker or nonexistent. When the absolute difference between two sets was fixed to 2, a more disparate ratio resulted in a higher accuracy. However, although nonsignificant, we did see an opposite trend on RT, with a more disparate ratio resulting in a slower RT. This finding is consistent with the pattern of results found in Experiments 1 and 2.

## Experiment 4: Spatial array with varying ratio and numerosity

To understand whether the effect of object numerosity in comparative number judgment was limited to objects shown in a temporal sequence or it could also be present in any comparative number judgment task, such as when objects are shown in a spatial array, in this experiment we presented all objects at once in a spatial array rather than showing them sequentially one at a time. As in the preceding experiments, we varied both the ratio and the numerosity of the two types of objects shown in a spatial array.

### Design and procedures

#### Participants

A total of 12 participants participated in the experiment (mean age, 24.33 ± 3.34 years; 9 females; 11 right handed). Of the 12 participants, 2 also took part in Experiment 1, 3 in Experiment 2, 1 in Experiment 3, 3 in both Experiments 1 and 3, and 3 in both Experiments 2 and 3.

#### Stimuli and design

The design of this experiment included the same 6 trial conditions as those appeared in Experiment 1. Specifically, the ratio between the two objects was set to be either 1:2 or 2:3, with the size of the smaller set to be either 2, 4, or 6, resulting in 6 possible trial conditions (2 ratios × 3 sizes for the smaller set).The trial conditions were, for the 1:2 ratio, 2 versus 4, 4 versus 8, 6 versus 12, and for the 2:3 ratio, 2 versus 3, 4 versus 6, and 6 versus 9. All objects for a given trial were shown all at once randomly intermixed on the screen for 200 ms. A gray dot then followed to cue the participants to decide which of the two object categories had more exemplars shown on that trial. As in Experiment 1, the key assignment was fixed for each participant and counterbalanced across participants. The objects appeared along two invisible concentric circles centered around the fixation. The inner circle had six possible object positions distributed evenly along the perimeter at a 60° interval starting from 1 o'clock, and the outer circle had 12 possible object positions along the perimeter at a 30° interval starting from 12 o'clock. The radii of the inner and outer circles subtended 4° and 10° of visual angle, respectively. Each object subtended 3° of visual angle.

#### Analysis

Following the same procedure as described in Experiment 1, a total of 2.06% of the data were removed as outliers from further analysis. All other details of the experiment were identical to those of Experiment 1.

### Results

**Figure 5.**
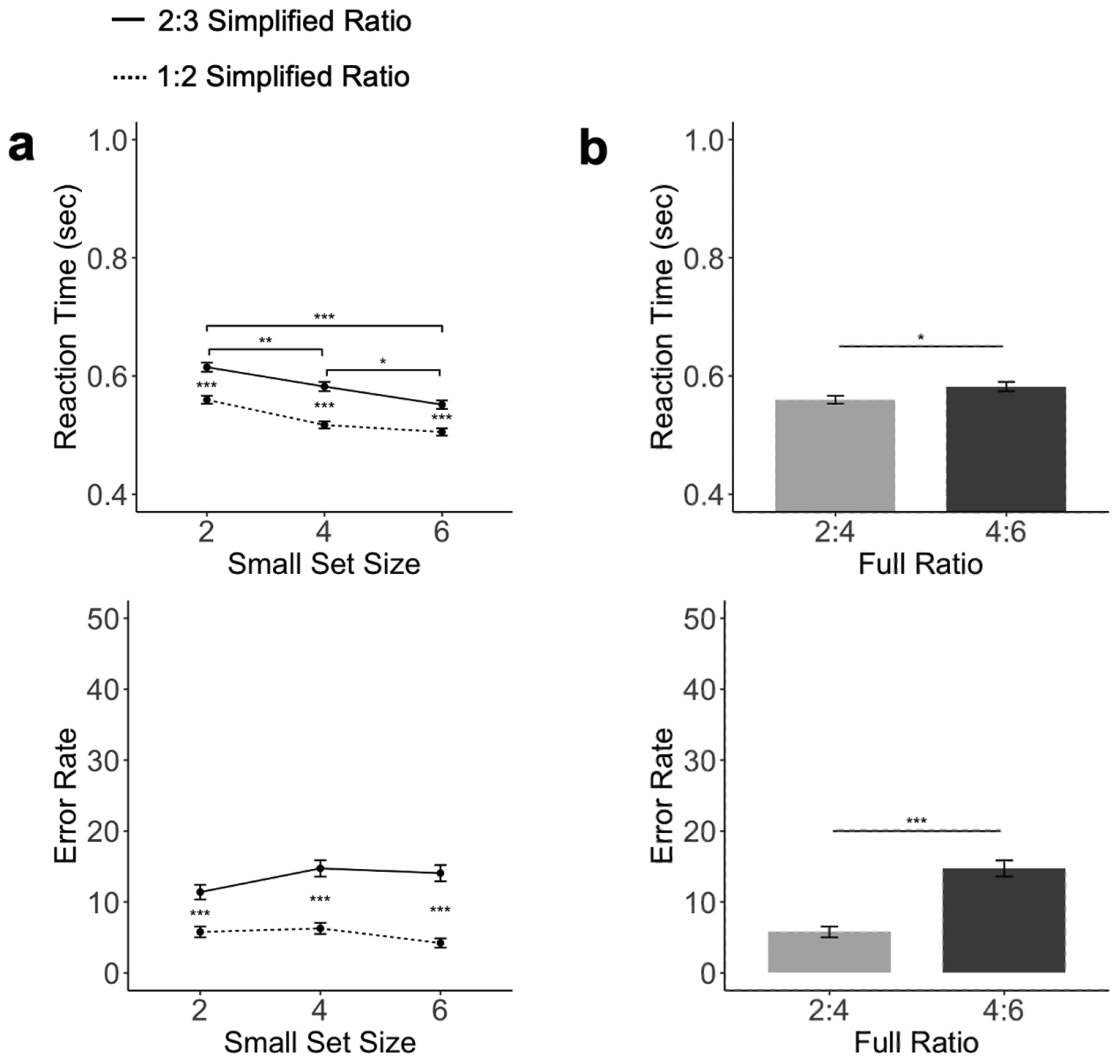
Results of Experiment 4. (a) RTs and error rates as a factor of ratio and the size of the smaller object set. (b) RTs and error rates when the absolute difference between the two object sets was fixed, comparing 2 versus 4 (at a ratio of 1:2) and 4 versus 6 (at a ratio of 2:3). The error bar represents ±1 standard error. *p < 0.05, **p < 0.01, ***p < 0.001, all corrected for multiple comparisons using the Benjamini–Hochberg method.

As before, we measured both the RT and error rates and the results are plotted by the number of objects shown in the small set (Figure 5a) and by the object ratio (Figure 5b). Also, see the mean and standard deviation for both RT and error rates for each full object ratio in Table 4.

**Table 4.** Results of Experiment 4. The mean (*M*) and standard deviations (SD) of RTs and error rates for each ratio (*N* = 12).

Full ratio	RT (*M*, s)	RT (SD, s)	Error rate (*M*, percent)	Error rate (SD, percent)
2:3	0.615	0.222	11.382	31.777
2:4	0.559	0.201	5.765	23.320
4:6	0.582	0.219	14.727	35.457
4:8	0.517	0.183	6.263	24.243
6:9	0.551	0.201	14.057	34.776
6:12	0.505	0.186	4.201	20.073

For RT measures, a repeated measures ANOVA revealed a significant effect of ratio, with faster RT for a more disparate ratio, *F*(1,11) = 37.35, *p* < 0.001, η^2^_*P*_ = 0.77, and a significant effect of numerosity, with faster RT when more objects were shown in a trial, *F*(2,22) = 9.33, *p* < 0.01, η^2^_*P*_ = 0.46 (Figure 5a). Post hoc paired *t*-tests for all trial pairs matching in ratio, but differing in numerosity were significant, with trials containing more objects having a faster RT, *t*s > 2.42, *p*s < 0.05, *d*s > 0.34 (corrected). There was no interaction between ratio and numerosity, *F*(2,22) = 0.30, *p* = 0.74, η^2^_*P*_ = 0.02.

As before, to understand if a difference in ratio still played a role when the absolute difference between the two sets was fixed, we also compared RT differences between 2 versus 4 (at a ratio of 1:2) and 4 versus 6 (at a ratio of 2:3). We found that 2 versus 4 had significantly faster RTs than 4 versus 6, the opposite of when items were presented sequentially, *t*(11) = 2.32, *p* < 0.05, *d* = 0.67 (Figure 5b).

For error rate measures, a repeated measures ANOVA revealed a main effect of ratio, with lower error rates for a more disparate ratio, *F*(1,11) = 83.05, *p* < 0.001, η^2^_*P*_ = 0.88, but no main effect of numerosity, *F*(2,22) = 0.96, *p* = 0.39, η^2^_*P*_ = 0.08 (Figure 5a). There was no interaction between ratio and numerosity, *F*(2,22) = 2.05, *p* = 0.15, η^2^_*P*_ = 0.02.

As in the RT analysis, we also found that when the absolute difference between the two sets was fixed to 2, 2 versus 4 had a lower error rate than 4 versus 6, *t*(11) = 8.84, *p* < 0.001, *d* = 2.55 (Figure 5b). Note that here the effect was in the same direction as the effect found in RTs.

Overall, these results showed that even when objects were presented all at once, both the relative number (ratio) and the absolute number (numerosity) of objects affected participants’ performance, with ratio having an effect on both RT and accuracy, and numerosity having an effect on RT but not accuracy. In other words, even in trials with the same ratio, having more objects greatly sped up the time it took to reach a correct decision. Unlike the previous three experiments, here when the absolute difference between two sets was fixed to 2, a more disparate ratio resulted in both a faster RT and a higher accuracy, showing that the effect of ratio was stronger than that of numerosity since the 4:6 set had a greater number of objects than the 1:2 set.

## Discussion

Although the neural and psychological mechanisms of perceptual decision-making have been studied for decades using largely sensory stimuli, such as dot motion and sound intensity, we presently do not understand fully the psychological factors that affect decision-making when there are two discrete, conflicting pieces of evidence shown in a temporal sequence and when more abstract visual information, such as object categories, were used. To address this, in this study, adapting the paradigms from Brunton et al. (2013) and De Lange et al. (2010) (see also Morcos & Harvey, 2016), we showed human participants a sequential presentation of objects drawn from two novel objects categories (Op de Beeck, et al., 2006) in Experiments 1 through 3. We asked participants to decide whether a given presentation contained more objects from one or the other object category. We measured the participants’ RT and error rate, and evaluated how ratio, object numerosity, and stimulus duration would impact performance. In Experiment 4, we presented all objects at once in a spatial array to further understand whether our findings may be replicated when objects are shown simultaneously rather than sequentially.

We found in Experiment 1 that, for sequential presentations of objects from two object categories, both a more disparate ratio (1:2 vs. 2:3) and greater numerosity of objects improved both RT and accuracy. That is, with a fixed object ratio, sequences with more total objects had lower RT and lower error rates than those with less total objects. In Experiments 2 and 3, we replicated these results. Moreover, we found that, when the total presentation duration was equated between trials containing the same ratio of objects but differing in numerosity, although a difference in accuracy was no longer present, a faster RT was still observed for trials with more objects. Such an RT benefit was not due to participants preparing their motor responses earlier in trials with more objects, because the same results were obtained when response key assignments were given after the presentation of all the objects. With all objects shown simultaneously in Experiment 4, with a fixed ratio, we still found an effect of object numerosity in RT (but not in accuracy), such that more objects in the display led to a faster RT. The latter result is consistent with prior work in ensemble statistics showing that estimations of mean tendency of a set are also faster and more accurate when more objects are shown (Robitaille, & Harris 2011).

Our results extend those of De Lange et al. (2010), which did not separately vary ratio and numerosity. Whereas De Lange et al. (2010) focused on understanding how the addition of each new piece of sensory information would impact behavioral performance and neural responses, because our goal here was to examine the contribution of object ratio and numerosity to performance, we averaged responses across all trial sequences sharing the same object ratio and numerosity. Our results from Experiment 1 showed that both object ratio and numerosity can affect both performance speed and accuracy.

In both our sequential and simultaneous displays, keeping the ratio the same, more objects and thus greater numerosity difference between the two sets led to a faster RT. This finding is consistent with the prediction of the drift-diffusion model in which a greater numerosity difference would result in more accumulated evidence toward one of the two object categories, resulting in a faster RT. However, when the same amount of sensory information was presented by showing twice the number of objects at one-half the presentation rate in a sequential display, we still found a significant decrease in the RT. There is, thus, a genuine benefit of numerosity that is separable from the total amount of sensory information accumulated.

Although an effect of ratio was expected for a comparative numerosity task, an effect of total numerosity was not based on Weber's law (Weber, 1834; Weber, 1978). In a recent study involving a sound intensity discrimination task, Pardo-Vazquez et al. (2019) found that although accuracy did not vary for the same intensity ratio, RT was faster when the intensity was higher. Our results were consistent with this finding in that greater object numerosity, which could be viewed as being equivalent to higher intensity, resulted in faster RT. However, unlike Pardo-Vazque et al. (2019), who found no effect in response accuracy, we found that trials with more objects also had lower error rates. This difference could be due to the sequential display used in our study. When we presented all objects at once in a spatial array (Experiment 4), we only found the effect of numerosity in RTs but not in accuracy, just like Pardo-Vazque et al. (2019). Thus, presenting objects sequentially, rather than simultaneously, resulted in an additional numerosity benefit in response accuracy.

When a sequence contained more objects, it had a longer total presentation duration. To understand if an effect of numerosity was the same as an effect of presentation duration, we equated both object ratio and presentation duration and found that the effect of numerosity was still present in RT but no longer in accuracy. Thus, when the total presentation duration was kept constant, an effect of numerosity in RT, but not in accuracy, was still present with a sequential presentation, similar to the effect obtained with a simultaneous presentation. This finding shows that the effect of numerosity is not the same as that of presentation duration.

But does total presentation duration also have an effect on performance? Brunton et al. (2013) reported that, with a fixed object ratio and numerosity, prolonged stimulus presentation duration in a sequential auditory discrimination task increased performance accuracy. In Experiment 2, in which the key assignment was fixed for each participant for the entire experiment, with the same object ratio and numerosity, we found that prolonged presentation duration improved response accuracy, but had no effect on RT. This finding is similar to the results of Brunton et al. (2013). In Experiment 3, when the key assignment was given at the end of a trial and varied from trial to trial, with the same object ratio and numerosity, there was no significant effect of presentation duration on response accuracy. However, a longer duration resulted in a slower RT. The inconsistencies across the two experiments warrant further investigation. Although not significant, in Experiment 3 there was a trend where a longer stimulus duration led to lower error rates, suggesting a potential speed–accuracy tradeoff, making it difficult to conclude whether or not an effect of duration would still benefit performance when such a tradeoff was absent. Note that Brunton et al. (2013) only reported response accuracy but not RT, leaving it unknown if a similar speed–accuracy tradeoff was present in that study. Thus, when both object ratio and numerosity were fixed, whether or not presentation duration alone may independently impact performance awaits further detailed investigation.

The working memory literature has long noted the capacity limitation of VWM for both simultaneously and sequentially presented arrays (e.g., Philips, 1974; Pashler, 1988; Luck & Vogel, 1997; Xu & Chun, 2006). It may be argued that attention and VWM could be more heavily taxed when more objects are shown in a temporal sequence, leading to a worse performance. However, the present results showed that more objects shown in a sequence actually benefited, rather than harmed, performance. This finding suggests that, rather than storing all the objects shown in a sequence, only the difference in numerosity between the two object sets may be stored. This finding is consistent with the numerosity effect we found, with a greater numerosity difference between the two sets of objects leading to a faster response time.

Can the effect of ratio reported here be accounted for entirely by the absolute difference between the two sets of objects? Here keeping the same absolute difference and varying ratio would require comparing high ratio object sets containing fewer total objects with low ratio objects containing more total objects. In our case, we would have to compare 2 versus 4 (1:2 ratio) with 4 versus 6 (2:3 ratio). In other words, such a comparison would pit ratio against numerosity. With the simultaneous presentation in Experiment 4, for the same absolute numerosity difference, a more disparate ratio resulted in faster RT and higher accuracy, demonstrating a stronger effect of ratio than numerosity and that the effect of ratio prevailed despite the same absolute difference between the two sets of objects. However, with the sequential presentation in Experiments 1 to 3, neither ratio nor numerosity dominated performance and the two effects seem to cancel each other. Specifically, Experiment 1 demonstrated a speed–accuracy tradeoff, such that, for the same absolute numerosity difference, a more disparate ratio resulted in a slower RT but greater accuracy. In Experiment 2, there was no significant difference in RT or accuracy for the two ratios, although opposite trends in RT and accuracy similar to those in Experiment 1 were observed. In Experiment 3, there was only a significant effect of accuracy, but an opposite trend in RT. With the absolute difference between the two sets of objects held constant, we thus saw a similar effect of ratio and numerosity, with numerosity showing a more pronounced effect of RT, whereas the ratio has greater influence on the error rate, albeit in the opposite direction.

One may argue that there is a confound of ratio and numerosity in the current design, such that, for all levels of the small set, the 1:2 ratio had a greater number of total objects than the 2:3 ratio: 2 versus 4 (6 objects, in total) is greater than 2 versus 3 (5 objects), 4 versus 8 (12 objects) is greater than 4 versus 6 (10 objects), and 6 versus 12 (18 objects) is greater than 6 versus 9 (15 objects). Thus, there remains the question of how the effect of ratio may be explained by total numerosity. Although our study was not designed to address this question, Brunton et al. (2013) fixed the total number of items shown sequentially while varying the ratio. They found that a higher ratio led to better performance. We take this as existing evidence that ratio is an important factor in sequential decision making. Indeed we did have comparisons in the current design that could dissociate the effect of ratio and numerosity. For example, if we look at 4 versus 8 (12 total) and 6 versus 9 (15 total) in Experiments 1 and 4, if only numerosity mattered, we should expect 6 versus 9 to have lower RT and error rate than 4 versus 8, but we found the opposite, showing that ratio plays a big role compared with numerosity in this case.

The focus of our study was to investigate the effect of object numerosity on decision-making. Hence we chose two easily discriminable ratios (1:2 and 2:3) to allow us to have a sufficient number of correct trials for both response RT and accuracy analyses. We found that the effect of numerosity was independent of ratios. Nonetheless, it would be interesting in future research to test if this holds for even smaller ratios such as 4:5 as well as for greater numerosities than those presented here (the greatest total number of objects shown was 18).

To conclude, we showed here that, for comparative numerosity judgment involving a sequential display, there was a benefit of numerosity, such that even with a fixed object ratio and fixed total presentation time, and with motor assignment varied from trial to trial, showing more objects led to faster decision performance.
